# Nomogram incorporating prognostic immune-inflammatory-nutritional score for survival prediction in pancreatic cancer: a retrospective study

**DOI:** 10.1186/s12885-024-11948-w

**Published:** 2024-02-12

**Authors:** Jie Yang, Hongkun Zhou, Huangbao Li, Fengqing Zhao, Kun Tong

**Affiliations:** Department of Hepatobiliary and Pancreatic Surgery, First Hospital of Jiaxing, Affiliated Hospital of Jiaxing University, 1882 Zhonghuan South Road, Jiaxing, Zhejiang 314000 China

**Keywords:** Pancreatic cancer, Prognostic immune-inflammatory-nutritional score, Survival, Nomogram

## Abstract

**Background:**

Prognosis prediction for pancreatic cancer has always been difficult in clinical practice because of its high heterogeneity and mortality. The aim of the study was to assess the value of prognostic immune-inflammatory-nutritional (PIIN) score on overall survival (OS) in postoperative patients with pancreatic cancer and to develop a nomogram incorporating PIIN score.

**Methods:**

This study retrospectively analyzed the clinic pathological data of 155 patients with pancreatic cancer who underwent radical surgery. PIIN score was calculated by measuring the fibrinogen (FIB), neutrophil to lymphocyte ratio (NLR), systemic immune-inflammation index (SII), albumin-bilirubin (ALBI) score, and prognostic nutritional index (PNI). Patients were divided into two groups by PIIN score levels over a threshold of 37.2. Univariate and multivariate analysis were performed using the Cox regression analysis model. The time-dependent receiver operating characteristic (ROC) curve was plotted to compare the prognostic values of the scoring systems. Finally, a nomogram based on PIIN score was constructed and validated.

**Results:**

Multivariate regression analysis showed that PIIN score (hazard ratio (HR) = 2.171, 95% confidence interval (CI) = 1.207–3.906, *P* = 0.010), lymphovascular invasion (HR = 1.663, 95% CI = 1.081–2.557, *P* = 0.021), poor tumor grade (HR = 2.577, 95% CI = 1.668–3.982, *P* < 0.001), bad TNM stage (I vs. II: HR = 1.791, 95% CI = 1.103–2.906, *P* = 0.018; I vs. III: HR = 4.313, 95% CI = 2.365–7.865, *P* < 0.001) and without adjuvant chemotherapy (HR = 0.552, 95% CI = 0.368–0.829, *P* = 0.004) were independent risk factors for OS. The time-dependent ROC curves revealed that PIIN score was better than the other scoring systems in predicting survival prognosis. And last, the nomogram established from independent factors such as PIIN score had good predictive power for OS. The ROC curve results showed that the AUC values for 1, 3 and 5 years were 0.826, 0.798 and 0.846, respectively. The calibration plots showed the superior clinical applicability of the nomogram.

**Conclusion:**

The nomogram model based on PIIN score can be utilized as one of the prognosis stratifications as well as postoperative follow-up for the development of individual treatment for pancreatic cancer.

## Introduction

 Pancreatic cancer is a common malignant tumor of the digestive system with high morbidity and mortality [1, 2]. Radical surgical resection is the most effective treatment for pancreatic cancer. However, early diagnosis of pancreatic cancer is difficult because its early symptoms are often non-specific. In addition, it is highly invasive and has a poor prognosis, with an overall 5-year survival rate of less than 11% [3]. Current options to predict the overall survival (OS) in pancreatic cancer remain unsatisfying. Because of the heterogeneous nature of pancreatic cancer, the treatment strategy and outcomes are diverse, even for tumors with the same TNM stage. Therefore, it is important to find more accurate predictive markers for the treatment of pancreatic cancer.

Pathological factors are widely recognized prognostic measures that can significantly reflect tumor phenotypic differences and have significant predictive power [4, 5]. However, many studies have shown that the outcomes of patients with cancer are determined not only by tumor-related factors but also by patient-related factors. Inflammation-related scoring systems, such as neutrophil to lymphocyte ratio (NLR) and systemic inflammation score, have been shown to correlate with prognosis for pancreatic cancer [6, 7]. Moreover, some scoring systems composed of immunity and nutrition-related markers could also predict the prognosis of pancreatic cancer, such as the prognostic nutritional index (PNI) and controlling nutritional status score [8, 9]. However, a single blood marker can not reflect the landscape of a patient’s immune function, nutrition status, and inflammation.

The prognostic immune-inflammatory-nutritional (PIIN) score is a new scoring system which includes all of the markers that have been predominantly used now. Recently, a retrospective study of 571 patients found that PIIN score could predict the prognosis in patients with resected intrahepatic cholangiocarcinoma, which helped surgeons identify high-risk patients and develop individualized treatment plans [10]. However, the significance of PIIN score in the prognosis of patients with pancreatic cancer has not been explored. Thus, this study aimed to investigate whether PIIN score is associated with survival after surgery in patients with pancreatic cancer.

## Patients and methods

### Patients

A total of 180 patients with resected pancreatic cancer between November 2011 and August 2022 at the Department of Hepatobiliary and Pancreatic Surgery, First Hospital of Jiaxing were retrospectively assessed. The inclusion criteria for this study were as follows: (1) pathologically diagnosed with pancreatic ductal adenocarcinoma, (2) patients received no prior anti-cancer treatment, (3) complete clinicopathological and follow-up data, and (4) radical resection performed with R0 margin. The exclusion criteria were as follows: (1) other concurrent malignancies, (2) active or chronic infectious or inflammatory status. (3) patients with distant metastasis, (4) tumor progression or death occurred within 1 month after surgery. In the end, 155 patients that met the criteria were included.

### Surgical methods and adjuvant therapy

Based on the location and size of the tumor, each patient underwent pancreaticoduodenectomy, distal pancreatectomy or total pancreatectomy, and routine dissection of the abdominal lymph nodes. Gemcitabine and fluorouracil combined with adjuvant chemotherapy were routinely administered to patients with pancreatic cancer, without contraindications, after radical surgery.

## Postoperative follow-up

In this study, overall survival (OS) was defined as the time between surgery and all-cause death or the last follow up. All patients were followed up every 3 months for 2 years after surgery, and the number of visits was reduced to every 6 months after 2 years. Survival data were extracted from outpatient or telephone records during follow-up. All patients were followed up until death or December 2022.

This study was conducted in accordance with the Declaration of Helsinki and was approved by the Research Ethics Committee of the First Hospital of Jiaxing (batch number: 2023-KY-670). We retrospectively used information about the participants’ previous clinical visits without direct contact with them and protected their privacy. The Ethics Committee of the First Hospital of Jiaxing approved the requirement for the waiver of informed consent for this study.

### PIIN score and other prognostic scoring systems

Data on the following clinical characteristics and clinicopathological information were obtained from electronic medical records of the hospital information system. Including: age, sex, tumor location, lymphovascular and nerve invasion, tumor size, grade, T stage, N stage, TNM stage, adjuvant chemotherapy, chemotherapy completion rate, and postoperative complications. Besides, lymphocyte count, neutrophil count, platelet count, fibrinogen (FIB), albumin, carbohydrate antigen 19 − 9 (CA 19 − 9) and bilirubin levels were determined within 7 days before surgery.

NLR = neutrophil count/lymphocyte count [11]. Systemic immune-inflammation index (SII) = platelet count × neutrophil count/lymphocyte count [12]. albumin-bilirubin (ALBI) score = -0.085× albumin (g/L) + 0.66 × log_10_ bilirubin (µmol/L) [13]. PNI = 10 × albumin (g/L) + 5 × lymphocyte count [14].

The PIIN score was calculated according to Jiang et al.’s method, with five parameters including NLR, SII, FIB, ALBI, and PNI [10]. PIIN score = NLR × 0.876 + SII × 0.0174 + FIB × 14.355 + ALBI × 2.209 − PNI × 0.386. In this study, we further adjusted the cut-off value using X-tile software. The ideal cut-off point was found to be 37.2 for PIIN score.

### Statistical analysis

In this study, chi-square test or Fisher’s exact probability method was used to assess the relationship between PIIN score and the clinicopathological features of the patients. Univariate and multivariate analyses were performed using Cox proportional hazards regression to assess the prognostic factors. The Kaplan-Meier method was used to draw the survival curve, and a parallel log-rank test was performed. A time-dependent receiver operating characteristic (ROC) curve and area under curve (AUC) were used to compare the prognostic abilities of PIIN score and other prognostic scoring systems. A nomogram was then built by using the variables in the multivariate analysis to predict OS at 1, 3, and 5 years after surgery. ROC curve analyses were performed to compare the predicting efficiency of the prediction model. Calibration curves were plotted to evaluate the consistency between predicted and observed survival. The cut-off values of age and CA 19 − 9 level were generated by the X-tile software (3.6.1; Yale University, New Haven, USA). Statistical significance was set at a two-sided *P*-value equal to 0.05.

SPSS (version 22.0; IBM Corporation, Armonk, NY, USA) and R software (R Project for Statistical Computing, Vienna, Austria) were used for data processing. The R-packet “timeROC” was used for time-dependent ROC curve analysis, and the nomogram was drawn using the R-packet “rms”.

## Results

### Relationships between PIIN score and clinicopathological characteristics

A total of 155 patients with pancreatic cancer were enrolled in this study according to the inclusion and exclusion criteria. Among them, 85 (54.8%) were males and 70 (45.2%) were females. Of the 155 patients, the median age was 66 years (IQR: 60.5–71 years); the median CA 19 − 9 level was 159.7 U/mL (IQR: 48.0-421.6 U/mL). As for tumor site, 107 (69.0%) tumors were primarily located in the head of the pancreas, while 48 (31.0%) tumors primarily occurred in the body or tail of the pancreas. According to the eighth edition of the American Joint Committee on Cancer (AJCC) staging system classification, 55 cases in stage I, 74 cases in stage II, and 26 cases in stage III. 89 cases (57.4%) received postoperative adjuvant chemotherapy. Postoperative complications occured in 43.9% of cases. The most common complications were related to infection (14.8%), followed by pancreatic leak (11.0%) and bleeding (5.8%).

Table 1 summarizes the relationships between PIIN score and clinicopathological characteristics. PIIN score was closely related to the tumor location (*P* = 0.003) and postoperative complications (*P* < 0.001). However, it had no significant correlation with age, sex, lymphovascular and nerve invasion, grade, tumor size, T stage, N stage, TNM stage, CA 19 − 9, adjuvant chemotherapy, type of adjuvant chemotherapy, chemotherapy completion rate, and type of postoperative complications (all *P* > 0.05).

**Table 1:** Relationships between PIIN score and clinicopathological characteristics in patients with pancreatic cancer

Project	PIIN score		χ^2^	P-value
	≤ 37.2	> 37.2		
Sex			3.443	0.064
Male	24(68.6)	61(50.8)		
Female	11(31.4)	59(49.2)		
Age (years)			3.425	0.064
≤ 63	18(51.4)	41(34.2)		
> 63	17(48.6)	79(65.8)		
Tumor location			8.853	0.003
Head	17(48.6)	90(75)		
Body/Tail	18(51.4)	30(25)		
Lymphovascular invasion			2.544	0.111
No	28(80)	79(65.8)		
Yes	7(20)	41(34.2)		
Nerve invasion			0.501	0.479
No	7(20)	18(15)		
Yes	28(80)	102(85)		
Grade			0.092	0.762
Well/moderate	15(42.9)	48(40)		
Poor/undifferentiated	20(57.1)	72(60)		
Tumor size (cm)			0.158	0.691
≤ 3	15(42.9)	56(46.7)		
> 3	20(57.1)	64(53.3)		
T stage			0.307	0.580
T1/T2	24(68.6)	88(73.3)		
T3/T4	11(31.4)	32(26.7)		
N stage			2.223	0.329
N0	21(60.0)	60(50)		
N1	13(37.1)	48(40)		
N2	1(2.9)	12(10)		
TNM stage			0.489	0.783
I	14(40.0)	41(34.2)		
II	15(42.9)	59(49.2)		
III	6(17.1)	20(16.7)		
CA 19 − 9 (U/ml)			1.731	0.421
≤ 91.2	15(42.9)	43(35.8)		
91.2-258.1	10(28.6)	28(23.3)		
> 258.1	10(28.6)	49(40.8)		
Adjuvant chemotherapy			1.272	0.259
No	12(34.3)	54(45)		
Yes	23(65.7)	66(55)		
Type of adjuvant chemotherapy			0.924	0.820
Gemcitabine	2(8.7)	10(15.2)		
Gemcitabine + Nab-Paclitaxel	5(21.7)	11(16.7)		
Tegafur	8(34.8)	25(37.9)		
Other chemotherapy regimen	8(34.8)	20(30.3)		
Chemotherapy completion rate			2.329	0.127
Complete	14(60.9)	28(42.4)		
Incomplete	9(39.1)	38(57.6)		
Postoperative complications			20.057	< 0.001
No	9(25.7)	78(65)		
Yes	26(74.3)	42(35)		
Type of postoperative complications			6.025	0.110
Infection	10(38.5)	13(31)		
Bleeding	4(15.4)	5(11.9)		
Pancreatic leak	9(34.6)	8(19)		
Other complications	3(11.5)	16(38.1)		

*Abbreviations*: *PIIN *prognostic immune-inflammatory-nutritional, *CA 19 − 9 *carbohydrate antigen 19 − 9

### Univariate and multivariate cox regression analysis

The results of the univariate analyses based on OS are shown in Table 2. Univariate analysis demonstrated that age, lymphovascular invasion, grade, N stage, TNM stage, adjuvant chemotherapy and PIIN score were significantly correlated with OS (all *P* < 0.05).

**Table 2:** Univariate analysis of poor prognostic factors for OS in patients with pancreatic cancer

Project	Univariate analysis		
	HR	95% Cl	*P-*value
Sex			
Male	Reference		
Female	1.116	0.750–1.660	0.589
Age (years)			
≤ 63	Reference		
> 63	1.602	1.054–2.433	0.027
Tumor location			
Head	Reference		
Body/Tail	0.761	0.488–1.185	0.227
Lymphovascular invasion			
No	Reference		
Yes	2.011	1.319–3.064	0.001
Nerve invasion			
No	Reference		
Yes	1.513	0.872–2.625	0.141
Grade			
Well/moderate	Reference		
Poor/undifferentiated	2.246	1.480–3.410	< 0.001
Tumor size (cm)			
≤ 3	Reference		
> 3	0.988	0.664–1.471	0.953
T stage			
T1/T2	Reference		
T3/T4	1.455	0.950–2.230	0.085
N stage			
N0	Reference		
N1	1.522	1.001–2.314	0.049
N2	4.016	2.040–7.904	< 0.001
TNM stage			
I	Reference		
II	1.571	0.986–2.502	0.047
III	3.388	1.896–6.055	< 0.001
CA 19 − 9 (U/ml)			
≤ 91.2	Reference		
91.2-258.1	1.365	0.795–2.343	0.259
> 258.1	1.938	1.214–3.094	0.006
Adjuvant chemotherapy			
No	Reference		
Yes	0.602	0.405–0.895	0.012
Chemotherapy completion rate			
Complete	Reference		
Incomplete	0.751	0.476–1.187	0.221
None	0.489	0.292–0.820	0.007
Postoperative complications			
No	Reference		
Yes	0.879	0.582–1.329	0.542
PIIN score			
≤ 37.2	Reference		
> 37.2	2.371	1.344–4.183	0.003

*Abbreviations*: *OS *overall survival, *HR *hazard ratio, *CI *confidence interval, *CA 19 − 9 *carbohydrate antigen 19 − 9, *PIIN *prognostic immune-inflammatory-nutritional

A Cox multivariate model was established to identify independent risk factors affecting OS. And PIIN score was identified as an independent factor to predict the OS (Table 3; hazard ratio (HR) = 2.171, 95% confidence interval (CI) = 1.207–3.906, *P* = 0.010). In addition, OS was markedly impaired among cases with lymphovascular invasion (HR = 1.663, 95% CI = 1.081–2.557, *P* = 0.021), poor tumor grade (HR = 2.577, 95% CI = 1.668–3.982, *P* < 0.001), bad TNM stage (I vs. II: HR = 1.791, 95% CI = 1.103–2.906, *P* = 0.018; I vs. III: HR = 4.313, 95% CI = 2.365–7.865, *P* < 0.001) and without adjuvant chemotherapy (HR = 0.552, 95% CI = 0.368–0.829, *P* = 0.004).

**Table 3:** Multivariate analysis of poor prognostic factors for OS in patients with pancreatic cancer

Project	Multivariate analysis		
	HR	95% Cl	*P-*value
Age (years)			
≤ 63			
> 63			0.107
Lymphovascular invasion			
No	Reference		
Yes	1.663	1.081–2.557	0.021
Grade			
Well/moderate	Reference		
Poor/undifferentiated	2.577	1.668–3.982	< 0.001
N stage			
N0			
N1			0.606
N2			0.888
TNM stage			
I	Reference		
II	1.791	1.103–2.906	0.018
III	4.313	2.365–7.865	< 0.001
Adjuvant chemotherapy			
No	Reference		
Yes	0.552	0.368–0.829	0.004
PIIN score			
≤ 37.2	Reference		
> 37.2	2.171	1.207–3.906	0.010

*Abbreviations*: *OS *overall survival, *HR *hazard ratio, *CI *confidence interval, *PIIN *prognostic immune-inflammatory-nutritional

### Prognostic value of the PIIN score and other prognostic scoring systems

**Fig. 1:**
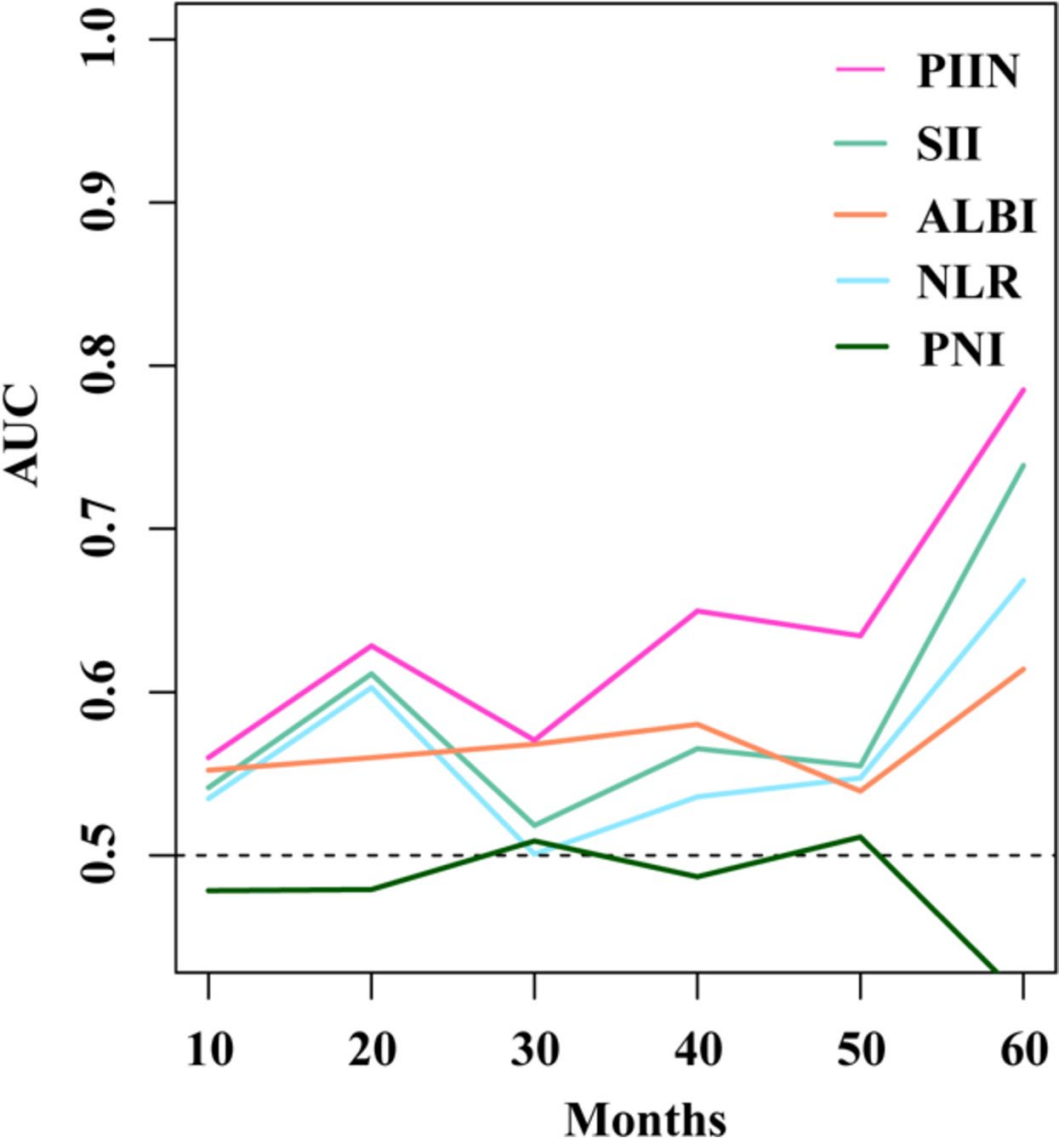
Comparison of the predictive accuracy of the different prognostic systems by the time-dependent ROC. The X-axis symbolizes the follow-up time, and the Y-axis represents estimated AUC for survival at specific time of interest

The time-dependent ROC curve was generated for the PIIN score and other prognostic scoring systems, and the AUC values were calculated at different time points. Time-dependent ROC curve analysis revealed that PIIN score was significantly superior to SII, ALBI, NLR, and PNI in predicting 1-, 3-, and 5-year OS (Fig. 1).

### Nomogram development and validation

**Fig. 2:**
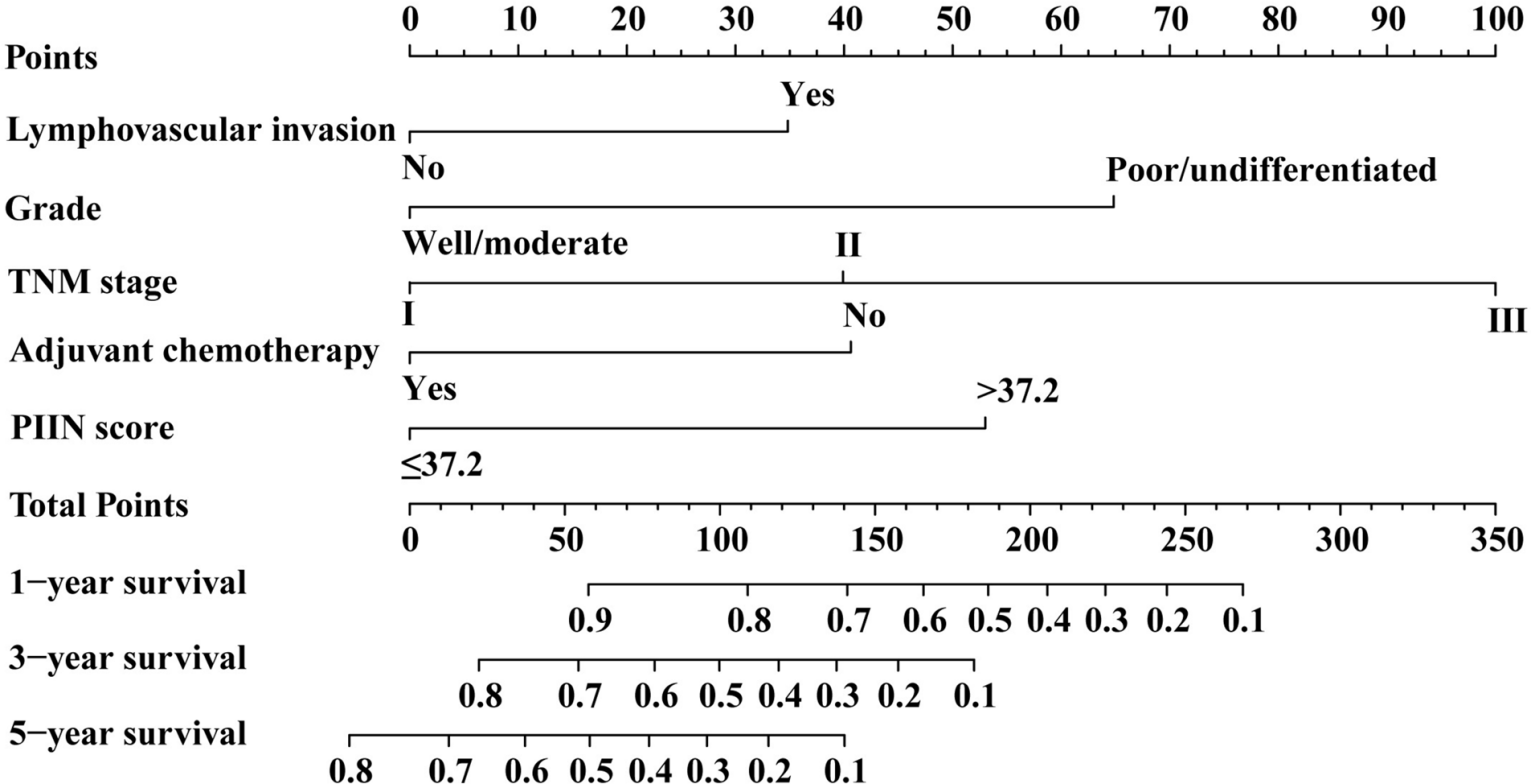
The nomogram based on PIIN score and clinical prognostic factors to predict the probabilities of 1-, 3-, and 5-year OS

Based on the results of univariate and multivariate COX regression analysis, independent prognostic factors were integrated into the construction of nomogram model to predict OS at 1-, 3-, and 5-year (Fig. 2).

**Fig. 3:**
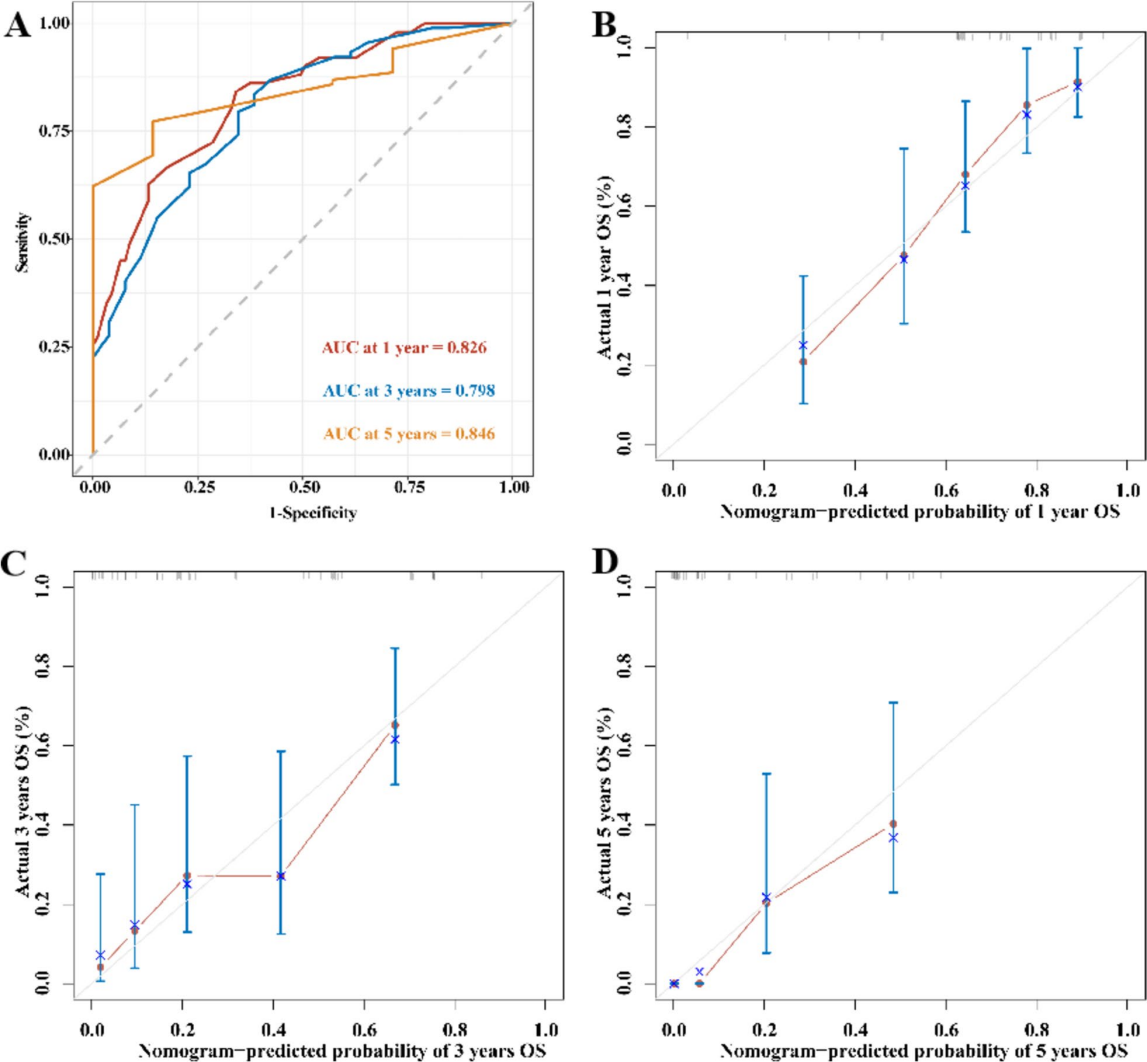
Evaluation of the nomogram model. **A** ROC curve of a predictive model that predicts 1-, 3-, and 5-year survival. **B**-**D** Calibration curves of prediction models for predicting 1-, 3-, and 5-year survival. The horizontal axis represents the nomogram-predicted survival, and the vertical axis symbolizes the actual survival. The curve in color closest to the 45° gray line gets the best prediction performance

The C-index value for patients with pancreatic cancer were 0.711 (95% CI: 0.676–0.746). The C-index was between 0.7 and 0.9 meant that the prediction accuracy of the model was high. The ROC curve results showed that the AUC values for 1, 3, and 5 years are 0.826, 0.798, and 0.846, respectively (Fig. 3A). The calibration curves for the probability of survival at 1, 3, and 5 years demonstrated good agreement between nomogram predictions and actual observations (Fig. 3B-D).

### Risk stratification based on the nomogram

**Fig. 4:**
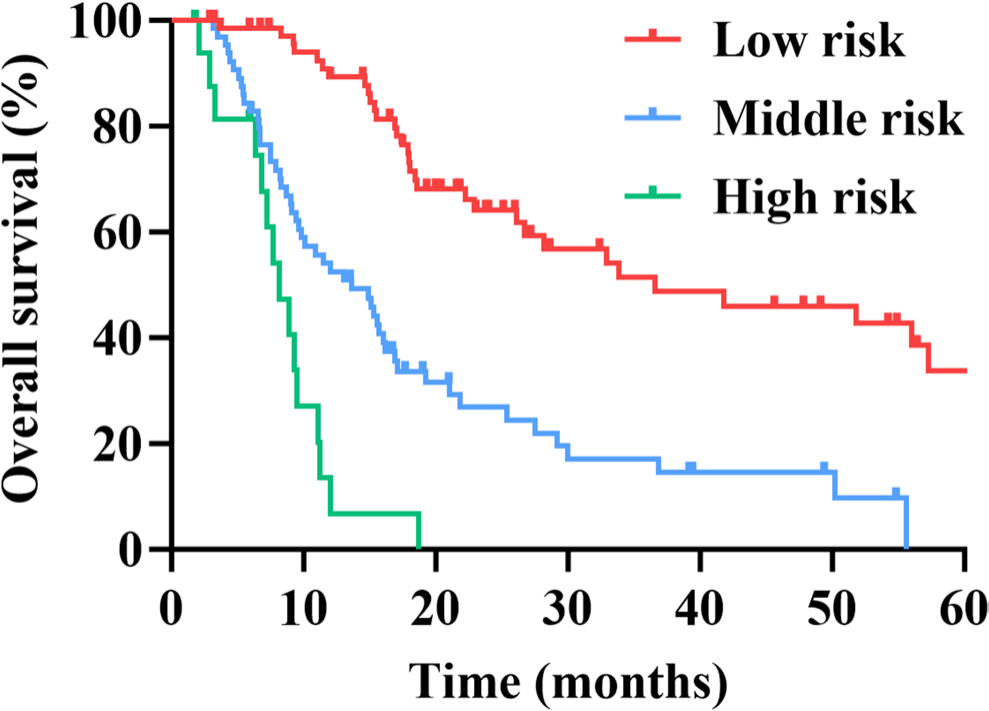
Kaplan-Meier survival curves of pancreatic cancer patients with different risks stratified by the nomogram

Based on different cut-off values of the total points determined by the X-tile software, we subdivided patients into low-, middle -, and high-risk groups, and applied Kaplan-Meier survival analysis to assess their survival. Patients were divided into low risk group (< 140), medium risk group (140–220) and high risk group (>220). The results of survival curve showed a significant difference in prognosis among the three risk groups (Fig. 4; *P* < 0.001). Therefore, the nomogram-based risk stratification system can significantly enhance the discrimination of survival of pancreatic cancer patients.

## Discussion

A single indicator is insufficient for prognosis risk stratification, highlighting the urgent need to integrate these markers. The PIIN score, which is a new scoring system that includes serum fibrinogen, NLR, SII, ALBI score, and PNI, comprehensively reflects the patient’s inflammatory, immune and nutritional status.

Previous studies have shown that postoperative complication was significantly associated with prognosis [15, 16]. A retrospective study by Aoyama et al. revealed that postoperative complication was associated with poor prognosis in patients with pancreatic cancer [17]. In our cohort, the most common complication was related to infection followed by pancreatic leak and bleeding. A previous study showed that poor nutritional status with low albumin level (< 3.5 g/dL) or low BMI (< 18.5 kg/m^2^) before surgery for pancreatic head cancer was a predictor of postoperative complications [18]. Similarly, PIIN score was markedly related to postoperative complications (*P* < 0.001). Therefore, PIIN score has great clinical significance for predicting postoperative complications and aiding the assessment of treatment tolerance.

Differences of the tumor location may also affect clinical and surgical outcomes. surgical margin positivity was more likely for tumors located in the uncinate process than for other tumors in a retrospective study [19]. Moreover, different surgical approach according to tumor location could have an impact on patient’s gastrointestinal function and delay in recovery to a different degree. In our study, a significantly higher PIIN score was observed in patients with pancreatic head cancer (*P* = 0.003). However, tumor location was not associated with the prognosis of pancreatic cancer. Larger clinical samples and prospective studies are needed to examine the relationship between prognosis and the tumor location.

Based on the prognostic analysis of 155 patients with pancreatic cancer who underwent radical surgery, we observed that PIIN score was an independent prognostic factor for OS. We compared the predictions of different prognostic scores by time-dependent ROC curve analysis. The results showed that PIIN score had the highest AUC value compared with NLR, SII, ALBI score, and PNI, and the prognosis prediction effect was the best. Thus, clinicians can better monitor patients considering the better accuracy of PIIN score in predicting prognosis at an individual level. Moreover, close follow-up and individualized adjuvant therapy after surgery are of great clinical significance for improving patient prognosis.

Systemic inflammatory status and local immune response in cancer patients are significantly associated with tumor progression and prognosis [20]. Tissue damage caused by chronic inflammation produces local anti-inflammatory cytokines that promote tumor cell proliferation [21]. As a major player in the innate immune system, neutrophils respond to various inflammatory signals, including cancer, and directly promote tumor progression, metastasis, and angiogenesis [22]. The secretion of cytokines such as IL-6 and IL-8 induces neutrophil recruitment to the tumor site and has pro-inflammatory and angiogenic effects [23–25]. Lymphocytes can be used to assess immune status, and they induce an antitumor immune response in the systemic circulation and tumor microenvironment to kill tumor cells [26]. Lymphocytopenia has been reported to be associated with prognosis of patients with pancreatic cancer [27]. Additionally, monocytes promote immune escape by limiting the infiltration of activated CD8 T cells into tumor microenvironment [28].

Pancreatic cancer is characterized by exocrine insufficiency and nutritional imbalance, which can lead to malnutrition and sarcopenia [29]. Cancer-related malnutrition makes patients more vulnerable to surgical injury and serves as a negative prognostic factor. Serum albumin is an important marker of host nutritional status and is closely correlated with the degree of malnutrition. In pancreatic cancer, PNI and Controlling Nutritional Status as the nutritional status indices not only reflect the overall nutritional status of the patient but also closely related to the prognosis [30, 31]. Therefore, carrying out a nutritional screening is a fundamental intervention in the diagnosis of pancreatic cancer and should be implemented at regular intervals during therapy.

Integrating multiple prognostic factors allows a more accurate evaluation of patients’ prognosis. In our study, the nomogram model based on the PIIN score can predict the prognosis of patients with pancreatic cancer after surgery. The nomogram excels in predictive performance, with ROC curves showing AUC values of 0.826, 0.798, and 0.846 for 1, 3, and 5 years, respectively. The calibration plots of the PIIN score-based nomogram model indicated that it has favorable discriminative ability in patients with pancreatic cancer. The developed nomogram not only comprehensively integrates numerous known clinicopathological features into a prognostic model but also expands the clinical applications of the PIIN score.

This study had some limitations. First, the present study, which lacked external validation, was a single-center cohort study, and selection bias might have affected the results. Second, the cut-off value of PIIN score needs to be further validated. In the future, prospective studies with larger sample sizes, and external validation of our findings in other populations, are essential.

## Conclusion

PIIN score is an independent prognostic factor for OS in patients with pancreatic cancer. A new nomogram prediction model based on PIIN score is established and validated, which can independently predict disease progression and survival of patients with pancreatic cancer that are undergoing surgical management

## Data Availability

The datasets generated during and/or analyzed during the current study are available from the corresponding author upon reasonable request.

## Abbreviations

OS: overall survival
NLR: neutrophil to lymphocyte ratio
PNI: prognostic nutritional index
PIIN: prognostic immune-inflammatory-nutritional
FIB: fibrinogen
CA 19 − 9: carbohydrate antigen 19 − 9
SII: systemic immune-inflammation index
ALBI: albumin-bilirubin
ROC: receiver operating characteristic
AUC: area under curve
AJCC: American Joint Committee on Cancer
HR: hazard ratio
CI: confidence interval
